# A Novel Treatment for Ewing’s Sarcoma: Chimeric Antigen Receptor-T Cell Therapy

**DOI:** 10.3389/fimmu.2021.707211

**Published:** 2021-09-10

**Authors:** Zili Lin, Ziyi Wu, Wei Luo

**Affiliations:** Department of Orthopaedics, Xiangya Hospital, Central South University, Changsha, China

**Keywords:** Ewing’s sarcoma, CAR-T therapy, solid tumors, immune targets, targeted therapy

## Abstract

Ewing’s sarcoma (EWS) is a malignant and aggressive tumor type that predominantly occurs in children and adolescents. Traditional treatments such as surgery, radiotherapy and chemotherapy, while successful in the early disease stages, are ineffective in patients with metastases and relapses who often have poor prognosis. Therefore, new treatments for EWS are needed to improve patient’s outcomes. Chimeric antigen receptor (CAR)-T cells therapy, a novel adoptive immunotherapy, has been developing over the past few decades, and is increasingly popular in researches and treatments of various cancers. CAR-T cell therapy has been approved by the Food and Drug Administration (FDA) for the treatment of leukemia and lymphoma. Recently, this therapeutic approach has been employed for solid tumors including EWS. In this review, we summarize the safety, specificity and clinical transformation of the treatment targets of EWS, and point out the directions for further research.

## Introduction

Ewing’s sarcoma (EWS), a malignant cancer of bones or soft tissues, occurs predominantly in children and young adults and is the second most frequent primary bone tumor after osteosarcoma. Traditional treatments, including aggressive neoadjuvant and adjuvant chemotherapy in combination with surgery and/or radiotherapy, have greatly improved the long-term survival of patients suffering from localized disease, with a 5-year survival rate of more than 70% (1–3). However, once the tumor cells have metastasized or recurred, patients often show poor outcomes (4), indicating the need for new treatments for EWS. To improve the efficacy and eliminate adverse side effects, such new therapies need to the following characteristics: 1) High specificity to the tumor lesions, often referred to as targeted therapy, which can reduce damage to normal tissues. 2) Efficacy for metastatic and recurrent tumor lesions. In the past decades, new immunotherapies have emerged, such as immune checkpoint blockers, therapeutic cancer vaccines, and so on (5, 6). Among them, immune checkpoint blockers and chimeric antigen receptor (CAR)-T cells meet the above requirements and have been widely used in researches and treatments of various cancers in recent years (7, 8). CAR-T therapy is a form of treatment that combines tumor specific antibody receptors with cytotolytic T cell activity (9). Excitingly, CAR-T therapy has been used to treat hematologic tumors with encouraging results (10, 11). Recently, CAR-T therapy has also been used for solid tumors (12, 13). Furthermore, research has focused on the application of CAR-T cells for primary bone tumors (14). Therefore, we attempted to summarize the recent knowledge accumulated on CAR-T cell therapy for EWS, so that researchers can have a comprehensive understanding of all aspects of this kind of therapy.

## Overview of CAR-T Cell Therapy

**Figure 1:**
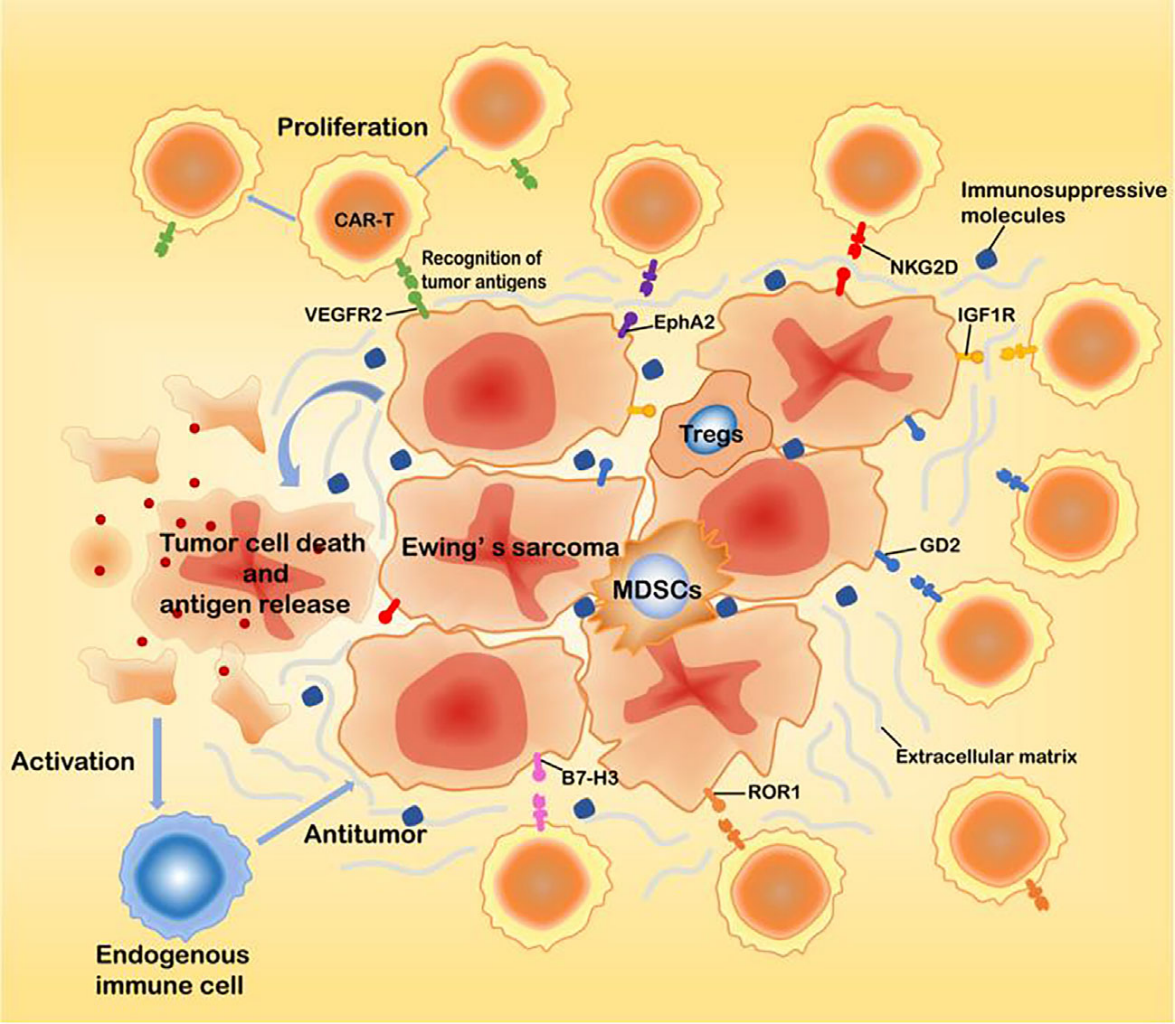
The main targets of CAR-T cells for EWS treatment.

Adoptive cell therapy (ACT) is a treatment strategy where immune cells with antitumor activity are introduced into a cancer patient (15). CAR-T cell therapy, a novel type of adoptive immunotherapy, has developed rapidly in recent years. CARs are engineered receptors composed of an extracellular single-chain variable fragment (scFv) derived from a monoclonal antibody, a transmembrane domain and an intracellular domain. The intracellular domains of CAR-T cells are usually derived from the T cell receptor CD3-ζ chain, which can bind to costimulatory molecules such as CD28 or 41BB (16). The first-generation CARs usually contain only the CD3-ζ chain signal transduction domain. The addition of one costimulatory molecule to the first-generation CARs resulted in the so-called second-generation CARs, while the third-generation CARs include the addition of two costimulatory molecules to first-generation CARs (17). This approach not only makes the immune cells have the targeting property but can also overcome the immune tolerance dilemma, such that the modified immune cells will have strong antitumor activity. In addition, CAR-based T cells therapies show potent antitumor activity without the limitation of traditional major histocompatibility complexes(MHC). After transplantation, CAR-T cells can proliferate in large numbers and exhibit long-lasting antitumor activity (11). The effective application of CAR-T cells in the treatment of tumors requires that the engineered CAR-T cells be specific to the tumor cells and equally lethal to metastases (18). Therefore, the pursuit of finding tumor-specific antigens is a highly important task. Currently, targets identified in EWS include the vascular endothelial growth factor receptor 2 (VEGFR2), type I insulin-like growth factor receptor (IGF1R), receptor tyrosine kinase-like orphan receptor 1 (ROR1), ganglioside2 (GD2), B7-H3 (CD276), hepatocellular receptor tyrosine kinase class A2 (EphA2), and natural-killer group 2D ligands (NKG2D ligands) (19–24)(**Figure 1**, **Table 1**). These receptors may serve as effective therapeutic targets for CAR-T cells to treat EWS and inhibit metastases.

**Table 1:** Targets of CAR-T in EWS.

	Characteristics	Expression in normal tissue	CAR-T examples	Possible ways to enhance efficacy	References
VEGFR2	A tyrosine kinase receptor	Expression in vascular endothelial cells	Only preclinical	Anti-B7-H3, Bispecific or polyspecific CARs	(20, 25–28)
IGF1R	A tetrameric transmembrane receptor tyrosine kinase	Widely distributed in normal tissues, such as myocardium, brain, bone and cartilage	Only preclinical	Bispecific or polyspecific CARs	(19, 26, 27, 29, 30)
ROR1	A tyrosine kinase receptor	Expression in normal tissue, highly in the gastric antrum and body	Only preclinical	Bispecific or polyspecific CARs	(26, 27, 31)
GD2	An N-acetyl neuraminic acid-containing glycolipid antigen	Low expression in normal tissue	Two ongoing clinical trials (NCT03356782, NCT03635632)	HGF-targeted neutralizing antibodies, EZH2 inhibitors, Bispecific or polyspecific CARs	(21, 26, 27, 32–34)
B7-H3	A member of the B7 family of immunoregulatory proteins	Low expression in normal tissue	Two ongoing clinical trials (NCT04483778, NCT04897321)	Anti-VEGF, Bispecific or polyspecific CARs	(25–27, 35)
EphA2	A tyrosine kinase receptor	Mainly confined to some epithelial cells	Only preclinical	Bispecific or polyspecific CARs	(26, 27, 36–38)
NKG2D	A powerful activating receptor expressed by NK/T cells	Expressed by NK cells and T cells	Only preclinical	Histone deacetylase inhibitors, Bispecific or polyspecific CARs	(26, 27, 39–41)

## Research on CAR-T Cells Targeting EWS Antigens

### VEGFR2 (Vascular Endothelial Growth Factor Receptor 2)

The vascular endothelial growth factor (VEGF) is an endothelial cell-specific mitogen that induces physiological and pathological angiogenesis. VEGF is a member of a larger family of growth factors that include VEGF-A, VEGF-B, VEGF-C, VEGF-D, and placental growth factors. The most frequently studied member of this family is VEGF-A, commonly known as VEGF, which has several distinct variants (VEGF_121_, VEGF_145_, VEGF_148_, VEGF_165_, VEGF_183_, VEGF_189_ and VEGF_206_). VEGF receptors (VEGFRs) include the three types of VEGFR1, VEGFR2 and VEGFR3, among which VEGFR2 plays a major role in regulating VEGF signaling in endothelial cells. The VEGF-mediated signaling pathway has been demonstrated to occur in tumor cells, and it plays a key role in tumorigenesis, including cancer stem cell function and tumor initiation (42, 43). Recently, there has been growing interest in the role of VEGF in EWS (44, 45). Surita Dalal et al. demonstrated that EWS secretes VEGF, and that the expression of VEGF is independently related to microvascular density, suggesting that VEGF may be the most important regulator of neovascularization in ESW. Moreover, Flk-1/KDR receptor tyrosine kinase inhibitors and anti-VEGF agents significantly inhibited EWS growth in the mouse model (46). In one study, researchers generated CAR constructs against both human and murine VEGFR2 to enable preclinical studies of the xenograft model of EWS. This study showed that VEGFR2-specific CAR-T cells effectively lysed VEGFR2-positive cells of the respective species and responded with potent antigen-specific degranulation responses, cytokine secretion, and proliferation (20). Thus, VEGFR2 is likely to be a suitable target for CAR-T cell therapy in EWS. VEGFR2-targeted CAR-T cells have been used in early clinical trials of metastatic melanoma and epithelial carcinoma (NCT01218867), but the respective clinical response has been poor. Therefore, to enhance the efficiency of VEGFR2-targeted CAR-T cells and translate them into clinical applications, further research on targeting the VEGF signaling pathway is essential to better serve EWS therapy. Several studies have reported that VEGF165 plays a significant role in EWS angiogenesis and tumor growth, and targeting VEGF165 can inhibit EWS growth (47–50). In addition, VEGF_165_ could promote osteolytic bone destruction in EWS (51). Therefore, it seems worthwhile to investigate whether targeting VEGFR and VEGF simultaneously increases the antitumor effect of EWS.

### IGF1R (Type I Insulin-Like Growth Factor Receptor)

IGF1R is a tetramer transmembrane receptor tyrosine kinase. The binding of ligand to the IGF1Rα subunit leads to the autophosphorylation of β subunit and the recruitment of adaptor proteins, ultimately resulting in the activation of signaling cascades that in turn contributes to proliferation, survival, transformation, metastasis, and angiogenesis (19, 29, 30). As IGF1R is expressed in EWS, many experiments have used it as an immune target for EWS treatment (52, 53). Various monoclonal antibodies have also been developed to treat EWS with a certain level of efficacy (54, 55). Thus, IGF1R-targeted CAR-T cell therapy for EWS appears to be a viable approach. According to a study by Xin Huang et al, IGF1R-targeted CAR-T cells showed specific cytotoxicity *in vitro* and mainly released IFN-γ, TNF-α, and IL-13 cytokines against sarcomas. These cells significantly inhibited sarcoma growth in both localized and disseminated pre-established sarcoma xenograft models. In addition, IGF1R-targeted CAR-T cells have also resulted in the benefit of prolonged survival in a localized sarcoma model (19). Although IGF1R-targeted CAR-T cells have a certain antitumor activity, it is not clear whether they have any toxic side effects on the body. Related studies have shown that IGF1R is also expressed in normal tissues (30). In a phase II study of EWS, researchers found that patients experienced adverse events such as neutropenia and leukopenia after treatment with ganitumab(a fully human anti-IGF1R antibody) (56). Xin Huang et al. reported that both lymphocytes and monocytes had low expression of cell-surface IGF1R, which made them not easily recognizable to IGF1R-targeted CAR-T cells (19). Off-target toxicity may be solved by the means of changing the affinity of CAR-T cells to the target or by adjusting the therapeutic dose of CAR-T cells. However, the systemic evaluation of off-target toxicity of IGF1R CAR-T cells should be performed before realizing their clinical application.

### ROR 1 (Receptor Tyrosine Kinase-Like Orphan Receptor 1)

Receptor tyrosine kinase orphan receptors 1 (ROR1) is one of the twenty different RTK families and is highly conserved in evolution. It consists of three distinct extracellular domains, including the immunoglobulin-like domain, cysteine-rich (CRD) and Kringle (KNG) domains, and the intracellular TK domain. The cytoplasm contains the TK domain with protein kinase activity, which is rich in serine, threonine, and proline motifs further downstream. ROR1 is not expressed in normal adult tissues, but is overexpressed in several human malignancies and may act as a survival factor for tumor cells (57). Experiments by Jenny Potratz et al. have shown that ROR1 is expressed in EWS cell lines and ROR1 silencing impairs EWS cell survival and migration (58). Moreover, Xin Huang et al. further demonstrated that ROR1 is highly expressed in sarcoma cell lines including EWS, osteosarcoma, rhabdomyosarcoma, and fibrosarcoma. Furthermore, the *in vitro* and *in vivo* anti-sarcoma activity of ROR1-targeted CAR-T cells were indicated (19). The safety of ROR1-targeted CAR-T cells was demonstrated in primates (59). However, it was recently shown that ROR1 expression is not specific to tumor tissue. Cell surface ROR1 has been observed in several areas of the parathyroid gland, pancreatic islet, and intestinal tract in humans, and it was particularly abundant in the stomach antrum and gastric body, although experiments in the macaque model have shown no significant adverse effects (31). Shivani Srivastava et al. designed a Logic-Gated ROR1 CAR that can save healthy tissues and target tumor cells, addressing the issue of off-target toxicity (60). In the “AND” gate of the logic-gated receptor, CAR-T cell activity only eliminates tumors that express both antigens A and B. Because the synthetic Notch receptor is specific for antigen A induced expression of CAR-specific antigen B, the “AND” logic gate can integrate multiple signals to regulate T cell function, thus allowing a more precise distinction between tumor tissue and normal tissue (61). Although the above studies have proved that ROR1-targeted CAR-T cells have a certain efficacy in treating EWS, ROR1-targeted CAR-T cells have not yet been subject to clinical trials, and the latest progress comes from an ongoing recruitment study (NCT02706392), revealing that it has great development potential.

### GD2 (Ganglioside2)

GD2, a cell surface molecule with a heavily restricted expression pattern, is highly expressed in EWS (62, 63). Due to the limited distribution of GD2 in normal tissues, it is safe for immunotargeting (32). S Kailayangiri et al. demonstrated that GD2 is expressed on the surface of EWS cell lines and primary EWS cells, and they proved that GD2-targeted CAR-T cells exert potent cytolytic responses against EWS cells (64). A different study also confirmed the antitumor activity of GD2-targeted CAR-T cells against EWS (65). However, other researches have shown that GD2-targeted CAR-T cells alone do not eliminate metastatic or orthotopically injected EWS cells. In fact, GD2-targeted CAR-T cells can prevent primary tumor growth and metastasis in EWS when combined with HGF-targeted neutralizing antibodies (21). In addition, Sareetha Kailayangiri et al. reported that the inhibition of enhancer of zeste homolog 2 (EZH2) enhanced the killing effect of GD2-targeted CAR-T cells against EWS (33). In a phase I study, researchers treated patients with neuroblastoma with GD2-targeted CAR-T cells and found no objective clinical response to treatment with GD2-targeted CAR-T cells alone (66). Therefore, the means to enhance the antitumor effect of GD2-targeted CAR-T cells is crucial to its successful clinical application. One strategy is to combine GD2 with other viable targets to construct T cells expressing multiple CARs. Another approach can combine GD2-targeted CAR-T cells with immune checkpoint inhibitors to improve efficacy.

### B7-H3 (CD276)

B7-H3 (CD276), a member of the B7 family of immunoregulatory proteins, is frequently overexpressed at high levels by solid tumor cells. B7 proteins bind to members of the CD28/CTLA-4 family which act as costimulatory signals in T cell activation (67). Moreover, B7-H3 is overexpressed during pathological angiogenesis, which may make it an attractive target for the selective destruction of tumor vasculature (68). Another study showed that B7-H3 may be a receptor expressed by cytotoxic lymphocytes inhibiting the activation thereof, and its deficiency or lack of inhibitive effect results in increased cytotoxic lymphocyte function in tumor-bearing mice (69). Taken together, the B7-H3 checkpoint may serve as a novel target for immunotherapy against cancer. In recent years, the use of B7-H3-targeted CAR-T cells for the treatment of solid tumors have received significant attention. In *in vitro* orthotopic and metastatic xenografts in mouse models of pancreatic ductal adenocarcinoma, ovarian cancer, and neuroblastoma, B7-H3-targeted CAR-T cells showed promising efficacy with no significant adverse effects (70). One study used indirect immunofluorescence to detect 8H9 (a monoclonal antibody targeting tumor-associated B7-H3) immunoreactivity in Ewing/primitive neuroectodermal tumor cell lines, in which two-third of samples were strongly positive and the rest were weakly positive, strongly supporting the presence of B7-H3 expression in EWS (71). The use of B7-H3 targets to produce CAR-T cells or antibodies for EWS treatment has also been attempted (72). Robbie GM et al. tried to use B7-H3-targeted CAR-T cells against pediatric solid tumors. They found that greater than 90% of the tested pediatric sarcomas expressed B7-H3 with high expression of EWS. Further experiments showed that B7-H3-targeted CAR-T cells can eradicate EWS xenografts *in vivo*, leading to a significant survival advantage compared to treatment control in mice (22). Therefore, B7-H3-targeted CAR-T cell therapy may be a viable option to treat EWS. Similar studies have shown that the inhibition or suppression of B7-H3 expression can increase the response of tumor cells to alkylation agents, drugs targeting DNA replication, PI3K/Akt/mTOR, and Ras/Rraf/MEK signaling inhibitors (73–76). Accordingly, the combination of B7-H3-targeted CAR-T cells with immune checkpoint inhibitors or traditional chemotherapy agents seems to be a feasible alternative. Chao Xie et al. reported that VEGF expression in tumor cells may be mediated by soluble B7-H3 (25), suggesting that the combination of anti-VEGFR and anti-B7-H3 or the construction of bispecific CAR-T cells embedded with VEGFR and B7-H3 may constitute a sound approach. The knowledge of the specific mechanism of B7-H3 in tumors will enable us to better treat B7-H3-positive tumors.

### EphA2(Hepatocellular Receptor Tyrosine Kinase Class A2)

Members of the Eph family are involved in cell transformation, metastasis, and angiogenesis. There are two classes of Eph receptor ligands: ephrin-A and ephrin-B. EphA2 is overexpressed in a variety of cancers, including breast cancer, melanoma, and prostate cancer (77). Associated studies have shown that EphA2 is upregulated in EWS cells, and participates in endothelial cell migration toward tumors to assist with tumor angiogenesis. Furthermore, EphA2 may be related to the aggressiveness of EWS (78–80). Therefore, blocking its function may be a promising method for EWS treatment. Kenneth Hsu et al. showed that EphA2-targeted CAR-T cells effectively killed EWS cells in mice, which was associated with prolonged survival. However, only a small percentage of EWS cells expressed EphA2 (23). It has been evidenced that EphA2 promotes EWS angiogenesis, tumor growth, and metastasis (79, 80), and that EphA2 CAR-T cells also exhibit antitumor activity in mice (23). Therefore, it is speculated that EphA2 CAR-T cells mainly influence EWS growth and metastasis by acting on tumor angiogenesis, which hypothesis clearly requires further experiments to test.

### NKG2D (Natural-Killer Group 2D)

NKG2D is a powerful activating receptor expressed by natural killer (NK) cells and T cells (39). In recent years, the role of NKG2D and its ligands in EWS have been the focus of increased attention (40, 81, 82). Moreover, interference with NKG2D expression may affect the efficacy of NK cells against EWS; activated NK cells have been shown to kill EWS cells with high efficiency (81, 83). The restoration of NKG2D receptor expression on immune effector cells may contribute to therapeutic strategies for EWS. Manfred Lehner et al. constructed NKG2D-specific CAR-T cells by lentiviral transduction or mRNA transfection, and these CAR-T cells effectively eliminated EWS cells *in vitro (* 24). On the one hand, to kill EWS cells more efficiently with NKG2D-specific CAR-T cells, we can modify T cells through the CARs editing technology. On the other hand, we can increase the efficiency by adding NKG2D ligands on tumor cells. Histone deacetylase inhibitors have been reported to upregulate the expression of NKD2G ligands in EWS (40, 41). Therefore, the combination of NKG2D-specific CAR-T cells and histone deacetylase inhibitors is considered as a recommended treatment for EWS.

### Other Potential Targets

Several additional targets have been found to be expressed in EWS, such as EGFR, CD99, PAPP-A, STEAP1 and endosialin, but these have not yet been used in CAR-T cell therapy for EWS (84–88). Furthermore, EGFR has been employed in a phase I clinical trial of metastatic pancreatic carcinoma (89). The above targets can all serve as potential targets for EWS treatment by CAR-T therapy.

## Discussion

Ewing’s sarcoma, an aggressive form of childhood cancer, is the second most common primary bone tumor. With the introduction of dose-intensive multiagent chemotherapy, the 5-year overall survival for the localized disease has improved to 70-75%, while the 5-year overall survival for metastatic or relapsed disease remains only at 20-30% (90, 91). Early hematogenous metastasis of EWS seriously affects the prognosis of patients, and the five-year survival rate of patients with metastasis is significantly lower than that of patients without metastasis (91, 92), which comprises the difficulty of EWS treatment. Traditional strategies including intensive chemotherapy fail to kill tumor cells completely in the blood, which are the key factors to EWS metastasis and recurrence (92). Targeted therapy and immunotherapy have shown some progress in EWS. Georgia J B McCaughan et al. reported an intensive pre-treated patient who had recurrent metastatic EWS achieved a clinical and radiological remission *via* PD-1 blockade (93), which is an encouraging result for the treatment of EWS. It is a pity that, there is little research in this field. For the treatment of EWS, there is still an urgent need for novel and effective treatments.

CAR-T cell therapy is a promising immunotherapeutical approach with encouraging results for tumors of the hematologic and lymphatic system (94). In comparison to traditional adoptive T cell therapy, editable CAR-T cells do not require MHC antigen presentation and can directly bind to target cell epitope for antitumor activity. Thus, CAR-T cells can overcome tumor escape and immune tolerance (95). Compared with checkpoint inhibitors, CAR-T cells can recognize lower levels of antigens, secrete cytokines that kill tumor cells, and self-proliferate to exert long-lasting antitumor effects (11, 96–99). Therefore, CAR-T cell therapy is clearly worthy of further studies. As EWS occurs more frequently in children and adolescents carrying more naive cells, and reports have suggested that adoptively transferred effector cells derived from naive T cells mediate superior antitumor effects, CAR-T therapy may exert superior antitumor effects in these EWS patients (100, 101). However, the adverse effects of CAR-T therapy on children and adolescents also should be focused. Currently, the main side effects of CAR-T therapy in children and adolescents remain the cytokine release syndrome and neurotoxicity, which can be prevented and treated by appropriate measures (102, 103). Furthermore, because the physiology of children and adolescents during growth is different from that of adults, it also should be discussed whether CAR-T cells therapy may have unique adverse effects. Therefore, more rigorous studies and clinical trials should be performed to explore the unique adverse effects of this therapy on children and adolescents. Excitingly, researches on CAR-T cells in the treatment of EWS have been pursued with certain encouraging developments. Currently, antigen epitopes used in CAR-T cells mainly include VEGFR2, IGF1R, ROR1, GD2, B7-H3, EphA2, and NKG2D. In most of the corresponding studies, researchers found that engineered targeted CAR-T cells exhibited antitumor activity in *in vitro* or *in vivo* preclinical models that were associated with a certain extent of extended survival. Nonetheless, these targets also have their limitations, such as off-target toxicity, insufficient effect and low expression, and they are still in their infancy, thus far from clinical applications. Therefore, more research is needed to address these issues before such targets can be translated into clinical practice. Regardless, CAR-T cell therapy for EWS is still worth undertaking. A considerable number of targets have been subject to clinical trials in solid tumor CAR-T cell therapy; even though their expression has not been established in EWS, targeted studies to address this gap can provide more options for EWS treatment by CAR-T, and are therefore worth conducting (104) (**Table 2**).

**Table 2:** Targets in clinical trials in solid tumor CAR-T therapy.

Antigen	Tumors	ID
HER2	Central nervous system tumor, pediatric glioma	NCT03500991
Nectin4/FAP	Nectin4-positive advanced malignant solid tumor	NCT03932565
EGFR806	Central nervous system tumor, pediatric glioma	NCT03179012
Mesothelin	Ovarian, cervical, pancreatic, lung	NCT01583686
Lewis Y	Advanced cancer	NCT03851146
LMP1	Nasopharyngeal	NCT02980315
FR-α	Ovarian	NCT00019136
EGFRIII	Glioblastoma and brain tumor	NCT01454596
Glypican-3	Liver	NCT02932956
PSCA	Lung	NCT03198052
MUC1	Advanced solid tumors, lung	NCT03179007, NCT03525782
IL-13Rα2	Glioblastoma	NCT02208362
MAGE-A1/3/4	Lung	NCT03356808, NCT03535246
gp100	Melanoma	NCT03649529
Claudin 18.2	Advanced solid tumor	NCT03874897
EpCAM	Colon, pancreatic, prostate, gastric, liver	NCT03013712
PSMA	Prostate	NCT01140373
AXL	Renal	NCT03393936
CD171	Neuroblastoma	NCT02311621
CD20	Melanoma	NCT03893019
MUC16	Ovarian	NCT02311621
DR5	Hepatoma	NCT03638206
c-MET	Breast, hepatocellular	NCT03060356, NCT03638206
CD80/86	Lung	NCT03198052
DLL-3	Lung	NCT03392064

On the whole, for CAR-T cells to be translated into clinical applications, the following issues need to be effectively addressed: 1. Certain targets are not tumor-specific and are present in normal tissue. For example, Balakrishnan A et al. reported that ROR1 is not only expressed in EWS and cell surface, but has also been detected in several areas of the parathyroid gland, pancreatic islet, and intestinal tract in humans, and is particularly abundant in the gastric antrum and body (31). 2.Although CAR-T cell therapy has shown encouraging results in the treatment of hematological malignancies, formidable obstacles limit its success in treating the vast majority of solid tumors, which may include antigen selection, tumor trafficking and the tumor microenvironment (TME) (105, 106). 3. The extensive side effects of CAR-T cells also need to be addressed, such as Cytokine Release Syndrome, or Immune Effector Cell-Associated Neurotoxicity Syndrome (6, 107).

The following measures can be adopted to solve the above problems: 1. Firstly, targets can be identified that are more specific to tumors. For instance, EGFRvlll has been found to express almost exclusively on tumor cells, but not in normal tissues, indicating that EGFRvlll is tumor specific (108, 109). Secondly, when the target is highly expressed in tumor cells and lowly expressed in normal cells, the damage to normal tissue can be reduced by controlling the therapeutic threshold. Thirdly, the affinity between CARs and cognate antigens can be adjusted to achieve targeting (110–112). Furthermore, an alternative approach is to design CARs targeting tumor-associated abnormal glycosylated glycopeptide epitopes (113–116). 2. The obstacles of CAR-T cell therapy, such as tumor penetration, resistance to killing, antigen escape and immunosuppression, can be addressed from the following aspects. 1) CAR-T cells can be engineered to express chemokine receptors to recognize upregulated chemokines in TME, thus increasing the infiltration of CAR-T cells (117, 118). In addition, T cell infiltration can be enhanced by designing CAR-T cells capable of degrading the extracellular matrix proteins that constitute the physical barrier to TME (119). 2) A combination of CAR-T cells with conventional therapies or immune checkpoint inhibitors may be worth exploring. For example, Christian Spurny et al. showed that, when antibodies were used to block HLA-G upregulation, it could help T cells to infiltrate EWS, thereby enhancing the antitumor activity of T cells (120). 3) Constructing immune cells expressing multiple CARs or combining multiple CAR-T cells may provide a higher efficacy in tumor cell destruction (26). Bispecific CARs have been designed for the treatment of hematological tumors (27). However, this approach may also increase toxicity to normal tissues and therefore requires rigorous evaluation for practical applications. 4) More co-stimulatory expression receptors can be introduced into CAR-T cells, and CAR-T cells can be constructed that target tumor antigens and immunosuppressive cytokines or immunosuppressive cells in TME to resist the tumor immunosuppressive effects on T cells (121, 122). 5) Currently, certain drugs can upregulate the low expression of antigen epitopes in tumors *via* epigenetics and increase the killing effect of CAR-T cells on tumors (33, 41, 106). As a whole, the treatment of solid tumors is not confined to the treatment of tumor cells themselves, and the role of TME should not be ignored. Since the TME can affect the infiltration of T cells towards the tumor, the immune escape of tumor cells and T cell exhaustion may occur (123). Therefore, the interaction between CAR-T cells and TME may be the key to the transformation of CAR-T into clinical applications, which prompts the need for urgent consideration. 3. With regards to adverse effects such as cytokine storm, targeted toxicity by CAR-T cells can be limited appropriately by the introduction of operations such as co-expression of suicide genes or inhibition of receptors (124). However, further and rigorous scientific studies are needed on CAR-T cells to rule out adverse effects.

## Conclusion

Therapies for EWS that involve CAR-T cells is a promising avenue, however, practicality and safety issues require further investigations.

## Author Contributions

WL selected the topic and revised the manuscript. ZL searched the literature, wrote the manuscript and figures, and ZW searched the literature. All authors contributed to the article and approved the submitted version.

## Funding

This work was funded by special funds for the construction of innovative provinces in Hunan Province (2020RC3058).

## Conflict of Interest

The authors declare that the research was conducted in the absence of any commercial or financial relationships that could be construed as a potential conflict of interest.

## Publisher’s Note

All claims expressed in this article are solely those of the authors and do not necessarily represent those of their affiliated organizations, or those of the publisher, the editors and the reviewers. Any product that may be evaluated in this article, or claim that may be made by its manufacturer, is not guaranteed or endorsed by the publisher.
